# Epidemiologic Responses to Anthrax Outbreaks: A Review of Field Investigations, 1950–2001

**DOI:** 10.3201/eid0810.020223

**Published:** 2002-10

**Authors:** Michael E. Bales, Andrew L. Dannenberg, Philip S. Brachman, Arnold F. Kaufmann, Peter C. Klatsky, David A. Ashford

**Affiliations:** * Centers for Disease Control and Prevention, Atlanta, Georgia, USA; † Emory University Rollins School of Public Health, Atlanta, Georgia, USA; ‡ Mt. Sinai School of Medicine, New York, New York

**Keywords:** anthrax, *Bacillus anthracis*, bacterial infections, disease outbreaks, public health, bioterrorism, Centers for Disease Control and Prevention (U.S.), historical article (publication type), zoonoses

## Abstract

We used unpublished reports, published manuscripts, and communication with investigators to identify and summarize 49 anthrax-related epidemiologic field investigations conducted by the Centers for Disease Control and Prevention from 1950 to August 2001. Of 41 investigations in which *Bacillus anthracis* caused human or animal disease, 24 were in agricultural settings, 11 in textile mills, and 6 in other settings. Among the other investigations, two focused on building decontamination, one was a response to bioterrorism threats, and five involved other causes. Knowledge gained in these investigations helped guide the public health response to the October 2001 intentional release of *B. anthracis*, especially by addressing the management of anthrax threats, prevention of occupational anthrax, use of antibiotic prophylaxis in exposed persons, use of vaccination, spread of *B. anthracis* spores in aerosols, clinical diagnostic and laboratory confirmation methods, techniques for environmental sampling of exposed surfaces, and methods for decontaminating buildings.

The intentional release of *Bacillus anthracis* in October 2001 greatly challenged the U.S. public health system. Collaborating with partners in other federal, state, and local health agencies, the Centers for Disease Control and Prevention (CDC) responded to these bioterrorism events by relying on experience investigating public health aspects of anthrax over the past 50 years (1). Topics addressed in these investigations included epidemiology, vaccines (2,3), controlling anthrax in industrial and agricultural settings (4), public health response to bioterrorism events (5), *B. anthracis* contamination of milk and meat (6), identifying *B. anthracis–*contaminated commercial products (7), decontamination methods for contaminated environmental sites, and laboratory methods, among others.

Field studies conducted by the Epidemic Intelligence Service (EIS) constituted the cornerstone of these investigative efforts (8). When invited by a state health department or national ministry of health, CDC’s EIS Officers conduct field investigations, Epidemic-Aids (known as Epi-Aids), in response to acute public health needs in the United States and other countries. Recently, historic documents from >4,000 Epi-Aids (approximately 90% domestic, 10% international) from 1950 to 1999 were made more accessible through the creation of an internal, searchable electronic database. It includes many unpublished CDC reports on early anthrax investigations, which form the basis of this report.

*B. anthracis*, the gram-positive, spore-forming, rod-shaped bacterium that causes anthrax (9), is most commonly a zoonotic pathogen. Human *B. anthracis* infections are rare in the United States; the number of cases has decreased steadily from an average of 35 reported cases per year in the 1950s to <1 reported case per year since 1980 (10,11) (Table 1). Most reported cases have been cutaneous. Before October 2001, the last case of inhalational anthrax in the United States occurred in 1976 (12,13).

**Table 1:** CDC field investigations of suspected anthrax in humans and animals and reported cases of anthrax in humans, United States, 1950–2001^a^

Years	Field investigations				No. of cases of anthrax in humans reported nationally^c^
	No. of investigations^b^	No. of human cases			
		Cutaneous	Inhalational	Total	
1950–54	2	1	0	1	223
1955–59	11	16	6	22	131
1960–64	4	5	1	6	54
1965–69	7	5	1	6	21
1970–74	8	4	0	4	13
1975–79	6	5	1	6	10
1980–84	0	0	0	0	2
1985–89	1	1	0	1	3
1990–94	1	0	0	0	1
1995–99	2	0	0	0	0
2000–01^d^	2	2	0	2	Not available
Total	44	39	9	48	458

^a^CDC, Centers for Disease Control and Prevention. ^b^Excludes three investigations of suspected anthrax conducted outside the United States (1967, 1986, 1998) and two investigations focused on decontamination of *Bacillus anthracis–*contaminated textile mills (1967, 1972). ^c^Sources: CDC. MMWR Summary of Notifiable Diseases, United States, 1994 (10); and MMWR Summary of Notifiable Diseases, United States, 1999 (11).  ^d^Before October 2001 bioterrorism-related anthrax cases.

To answer questions raised when the bioterrorism-related cases of anthrax were identified in October 2001, we reviewed results of field investigations of anthrax. We also identified current questions for which past experience with anthrax provided relatively little information and for which further research is needed.

## Methods

CDC anthrax-related field investigations from 1950 to 2001 were identified from several sources. First, the new database of historical Epi-Aid documents (1950–1999) was searched to retrieve all documents in which “anthrax” or “anthracis” appeared either as an assigned keyword or as a text string in a full-text search. Epi-Aid documents related to anthrax investigations in 2000 and 2001 were identified manually in an EIS administrative database. These searches identified a variety of types of documents, including initial requests for epidemiologic assistance, interim progress reports, final reports, and memoranda.

To identify published reports on these Epi-Aid investigations, we searched indexes to the Morbidity and Mortality Weekly Report (MMWR) for anthrax-related reports for the years 1961–2001. The individual issues of MMWR and its predecessor (Weekly Morbidity Report) were searched manually for the years (1950–1960) for which no index exists. To identify published reports on anthrax-related Epi-Aid investigations, we searched Medline for the years 1966–2001 and Index Medicus for 1950–1965. The names of the lead investigators from the Epi-Aids were used as keywords.

Additional CDC anthrax-related field investigations were identified by two coauthors (PB and AK) who were personally involved in most anthrax investigations conducted by the agency since the 1950s. References describing these additional investigations were located in the MMWR and in published medical articles. To limit this report to a description of CDC’s institutional experience, rather than a broader review of publications on anthrax investigations, we excluded (a) anthrax case reports published in the MMWR but unrelated to a CDC field investigation and (b) published reports on anthrax by investigators not affiliated with CDC.

From the unpublished Epi-Aid documents and published reports for each investigation, we abstracted the following information: year, location, number of human and animal cases, clinical form of the disease, occupational or other exposures for human patients, environmental sampling methods and data, and study recommendations.

## Results

A total of 49 relevant field investigations (Table 2) were included in this report: 42 Epi-Aids and 7 other investigations. Detailed reports and MMWR published summaries were available for 39 (93%) of the 42 Epi-Aid field investigations. For three agriculture-related investigations (Epi-Aids 1963-2, 1959-38, 1957-17), only the initial invitation for epidemiologic assistance was available for review.

**Table 2:** Characteristics of CDC field investigations of anthrax in humans and animals, 1950–August 2001^a^

Year	Location	No. of cases		Reference	Comments
		Human	Animal		
Agricultural settings (n=24 investigations)				
2001	TX (southwest)	1	1,638	Epi-Aid 2001-61	Large epizootic affecting 63 properties in five counties; members of at least 11 animal species were infected with *Bacillus anthracis.*
2000	ND (east)	1	Multiple	Epi-Aid 2000-69, (14)	USDA recommended quarantine on affected premises, vaccinating livestock on surrounding premises, and burning and/or burying infected carcasses, bedding, and other nearby materials.
1998	Kazakhstan	At least 53	Multiple	Epi-Aid 1998-83	Multivariate analysis found highest risk for cutaneous anthrax from slaughtering, butchering, and cutting *B. anthracis*–infected animals; eating cooked infected meat not an important risk factor.
1998	Uvalde, TX	One vaccine exposure	0	Epi-Aid 1998-55	Patient accidentally exposed to attenuated live anthrax vaccine while vaccinating horse, experienced severe myalgia and fatigue, then began antibiotic prophylaxis and recovered. Laboratory tests negative for *B. anthracis*.
1993	ND (southeast)	0	8	(15)	NIOSH and USDA investigation following major flooding, anthrax in livestock, and soil contamination. Concern over contaminated water supply, but all water samples negative.
1986	Paraguay	At least 21	0	Epi-Aid 1986-39, (16)	Community outbreak of cutaneous anthrax in a remote village.
1979	Clay County, IA	0	16	Epi-Aid 1979-95	Raising chlorine level to 2 ppm eliminated two positive samples in well water. In local hospital records, no difference in number of gastrointestinal symptoms compared with same month in previous year.
1976	Foard and Cottle Counties, TX	0	At least 160	Epi-Aid 1976-115, (17)	Significantly higher attack rates in bulls and horses; evidence against flies as important vector.
1974	Falls County, TX	0	At least 236	Epi-Aid 1975-6, (18,19)	*B. anthracis–* positive sample from city water tap, so city water supply was hyperchlorinated. Soil samples collected to document efficacy of carcass incineration were negative.
1971	Danville, PA	0	33	Epi-Aid 1972-19	*B. anthracis* isolated from both hay and soil samples.
1971	Gonzales, LA	2	588	Epi-Aid 1971-131, (3,20,21)	One culture positive and one negative in exposed veterinarians. Low attack rate in calves reduced likelihood that biting flies were an important vector.
1970	Yoder, WY	0	8	Epi-Aid 1971-44, (22)	Veterinarian placed on antibiotic prophylaxis as a result of laceration while performing necropsy.
1968	Inyo County, CA	1	176	Epi-Aid 1969-20, (23)	Extensive discussion and literature review of *Tabanid* species (horsefly) as potential vector; role in transmission remains inconclusive.
1968	Hampton, CT	0	3	Epi-Aid 1968-78	204 kg of *B. anthracis–*contaminated meat sold as hamburger before investigation. No human cases of anthrax known to have occurred as a result.
1965	Grand Forks, ND	0	19	Epi-Aid 1966-12, (24)	30 diabetic children swam 3 miles downstream from where an animal was found dead from anthrax; riverborne spread determined minimal; prophylaxis considered unnecessary.
1962	MS	0	Multiple	Epi-Aid 1963-2	Involved many counties.
1959	Brownsville, Cameron County, TX	5	125	Epi-Aid 1960-12	Two cases laboratory confirmed. Cases occurred in three veterinarians and two other patients who had intimate contact during necropsy, handling, or skinning.
1959	NJ (south)	1	2 cows, many hogs	Epi-Aid 1959-38	Not laboratory confirmed. Several hogs developed illness after feeding on entrails of sick cows.
1958	LA (north)	0	15–20	Epi-Aid 1958-42	Involved cows, sheep, and horses.
1957	Vinita, OK	1	400–500	Epi-Aid 1958-11, (25)	Large epizootic on farms curtailed after intensive immunization campaign.
1956	Saratoga, WY	0	Multiple	Epi-Aid 1957-17	Animal anthrax in mountainous area led to concern over water supply downstream.
1956	MS (northwest)	0	>250	Epi-Aid 1957-3	No evidence to support insectborne transmission, despite local beliefs. Involved 224 head of cattle, 42 mules, 5 horses, 3 sheep, 2 goats, multiple hogs. One case of suspected anthrax in a child was investigated and determined to be mumps.
1955	LA (southeast)	0	1,404	Epi-Aid 1955-5	Large epizootic in cattle. Unconfirmed reports of four human cases. *B. anthracis* isolated from flies in two instances at State Animal Disease Laboratory.
1952	OH (five counties)	0	Multiple	Epi-Aid 1952-13, (26)	*B. anthracis* isolated from swine feed; contaminated bonemeal suspected as source of infections.
Textile mills (n=13 investigations)				
1987	Charlotte, NC	1	0	Epi-Aid 1987-77, (27)	Suspected cross-contamination of Australian wool from storage space shared with contaminated West Asian cashmere.
1978	NH (southeast)	2	0	Epi-Aid 1978-65	Patients did not wear protective equipment. One had systemic signs and symptoms (fever, headache, sore neck, malaise, anorexia) after his initial lesion was lanced. Subsequent full recovery.
1978	Shelby, NC	2	0	Epi-Aid 1978-47	Contents of vacuum cleaner bags or floor sweepings from four employee homes were collected; 1 tested positive for *B. anthracis*. 300 soil samples tested from mill premises, landfill site, and nearby residences*.* In mill, more positive samples in rooms where earliest processing occurred.
1974	Belton, SC	1	0	Epi-Aid 1974-77	Report suggested prevention should be based on minimizing contact between employees and contaminated material, and on routine vaccination of employees at risk. Patient not adequately vaccinated.
1972	Manchester, NH	N/A	N/A	Epi-Aid 1972-94	Effectiveness of formaldehyde vapor decontamination of *B. anthracis* spores assessed using spore strips in treated and untreated (control) areas of mill complex, and comparing pre- and posttreatment surface samples. No positives among 599 posttreatment specimens.
1967	Dillon, SC	N/A	N/A	(28)	A building contaminated with *B. anthracis* was successfully decontaminated with formaldehyde vapor. 100,000 spores on 24 plates pretreatment were reduced to 21 sterile plates, and 3 plates with 2 colonies each, posttreatment. 26 of 142 surface swabs tested positive before decontamination, and 1 of 200 swabs tested positive 6 months after decontamination. Building was deemed safe for occupancy and no further cases were reported.
1966	Manchester, NH	2	0	Epi-Aid 1967-43	Patient with inhalational anthrax had history of "smoker's cough," diabetes, alcoholism, and chronic pancreatitis. Exposure believed to have occurred while patient worked for 4–5 hours directly opposite a goat hair–processing mill.
1961	Philadelphia, PA	1	0	Epi-Aid 1961-40; (29)	After case reported, supplies of new and improved Wright vaccine sent to mill for use among employees.
1960	SC	4	0	Epi-Aid 1960-31, (30)	All four cases responded well to antibiotic treatment.
1957	Philadelphia, PA	1	0	(31,32)	Two additional inhalational cases mentioned that occurred over an 8-year period in persons living near the same contaminated tannery.
1957	Manchester, NH	9	0	Epi-Aid 1958-18, (33–36)	Employees noted increased dust in air after initiating a new scouring technique in textile mill.
1956	Monroe, NC	>5	0	Epi-Aid 1956-29, (37)	Studies indicated heavy environmental contamination of mill with *B. anthracis* spores.
1953	Monroe, NC	1	0	Epi-Aid 1953-14	Nasal swabs of employees performed to assess exposure. No results available.
Other settings (n=7 investigations)				
1998	CA, IN, KY, TN	0	0	Epi-Aid 1999-25, (38)	Evaluation of multiple telephone threats and letters alleged to contain *B. anthracis*. Report included recommendations for response to bioterrorism threats.
1976	Morro Bay, CA	1	0	(12,39,40)	Suspected source of anthrax in home craftsman was contaminated yarn imported from Pakistan. Multiple samples of yarn tested positive for *B. anthracis.* Subsequent CPSC warning on imported yarn.
1975	Camden, NJ	3	0	(41–43)	Cutaneous anthrax in three gelatin manufacturing plant workers from contact with contaminated dry cattle bones; FDA recall of dicalcium phosphate animal feed product.
1974	Sequim, WA	0	42	(44,45)	Several cougars and other large felines on private game farm died after feeding on infected horsemeat. Primary source: horse's saddle pad contained *B. anthracis–*contaminated goat hair from Afghanistan and Pakistan. Subsequent CPSC warning on contaminated saddle pads.
1974	Haiti; FL	1 in US; 194 in Haiti (1963-1974).	0	Epi-Aid 1974-96, (7,46–50)	One human case in U.S.; 194 cases identified in Haiti in 1963–1974. 72 (25%) of 287 Haitian goatskin handicrafts tested from January to May 1974 were culture positive for *B. anthracis*, including voodoo balancing dolls, rugs, whole skins, mosaic pictures, purses, and drums. Subsequent CPSC warning on contaminated Haitian goatskin products.
1966	Manchester, NH	1	0	Epi-Aid 1967-43-3	Source of cutaneous infection in housewife unknown, but knitting yarn could not be ruled out. Three samples from knitted sweater positive for *B. anthracis*; samples from other sources negative.
1964	Oxford, OH	1	0	(51,52)	Fatal cutaneous anthrax in installer of pipe insulation made with imported goat hair. Insulation and goat hair samples tested positive for *B. anthracis*.
Suspected anthrax shown due to other causes (n=5 investigations)				
1975	Yavapai County, AZ	1	0	Epi-Aid 1975-115	23-year-old male machinist initially thought to have anthrax but quickly determined to have plague.
1969	Casper, WY	1	0	Epi-Aid 1969-78	Meat packing company employee; anthrax thought not to be responsible.
1967	Nepal	26	Multiple	Epi-Aid 1968-34	Community outbreak of cutaneous disease; subsequently diagnosed as plague.
1965	Charleston, SC	1	0	Epi-Aid 1966-18, (53)	Cutaneous disease in customs inspector; *B. anthracis* not implicated.
1957	Jamestown, NY	5	0	Epi-Aid 1958-16	Cutaneous disease in butchers; later believed to be a streptococcal or staphylococcal infection.

^a^CDC, Centers for Disease Control and Prevention; CPSC, Consumer Product Safety Commission; OSHA, Occupational Safety and Health Administration; FDA, U.S. Food and Drug Administration; USDA, U.S. Department of Agriculture; NIOSH, National Institute for Occupational Safety and Health.

Of these 49 field investigations, 41 (84%) involved human or animal infections with *B. anthracis*, 2 were evaluations of decontamination of *B. anthracis–*contaminated textile mills (1967 and 1972), and 1 was an investigation of bioterrorism threats involving anthrax (1998). In the other 5 investigations, *B. anthracis* was not found to be the causative organism, despite initial suspicion. Because these investigations include only anthrax cases for which CDC’s assistance was requested, they represent only a small proportion of the total number of U.S. cases reported during this period (Table 1).

Most of the investigations (41/49, 84%) were conducted from 1950 to 1980; only 8 anthrax-related investigations were conducted by CDC from 1980 until the October 2001 bioterrorism events. This trend mirrors the decline in reported U.S. anthrax cases in the latter half of the 20th century (10) (Table 1).

### Site

Among the 41 field investigations involving infection with *B. anthracis* (Table 2), 24 involved an agricultural setting (farms, contact with livestock, or both), 11 textile mills, 4 *B. anthracis–*contaminated commercial products, and 1 contaminated cow bones; in 1 instance, the source of infection was not determined. Thirty-eight (93%) of the 41 investigations took place in the United States; other investigations were conducted in Haiti (1974), Paraguay (1986), and Kazakhstan (1998).

### Clinical Form and Mechanism of Infection

All U.S. investigations involved cutaneous or inhalational anthrax (Table 3). Excluding large outbreaks in Kazakhstan and Paraguay, investigations in this report include 39 cutaneous and 9 inhalational cases of human anthrax. Among the investigations with available information on age and sex of patients, ages ranged from 19 to 67 years (median 40 years), and most cases were in males (Table 3).

**Table 3:** Inhalational and cutaneous anthrax in humans in CDC field investigations, United States, 1950–2001^a^

Year	Location	Occupation	Source	Age, sex	Reference^b^	Comments
Inhalational (N=9 cases)						
1976	Morro Bay, CA	Self-employed weaver	Imported yarn	32, M	(12)	Fatal inhalational anthrax due to contaminated imported yarn containing goat hair.
1966	Manchester, NH	Metal shop employee	Nearby mill processing goat hair	46, M	Epi-Aid 1967-43	Dust from neighboring goat hair mill identified as source. Incidence of anthrax at plant decreased with mandatory vaccination. Patient's coexisting illnesses may have contributed to susceptibility.
1961	Philadelphia, PA	Secretary in textile mill	Goat hair	50, F	Epi-Aid 1961-40	Fatal inhalational anthrax. Unusual because little contact with goat hair in routine work duties.
1957	Manchester, NH	Gillboxer in textile mill	Goat hair	60, M	Epi-Aid 1958-18	Five inhalational cases of anthrax (four fatal) occurred in the 600 employees of a textile mill. Four cutaneous cases occurred during the same outbreak.
		Bobbin cleaner and weaver		65, F		
		Card fixer		49, M		
		Card tender		61, M (recovered)		
		Noil remover		33, M		
1957	Philadelphia, PA	Factory employee	Nearby mill processing goat hair	29, M	(31,32)	Fatal inhalational anthrax in man with sarcoidosis. Possible exposures from glue made from animal hides, or goatskin tannery with sweepings and surfaces testing positive for *Bacillus anthracis*, which patient walked by daily.
Cutaneous^c^ (n=39 cases)						
2001	TX (southwest)	Farm worker	Infected animal	?, M	Epi-Aid 2001-61	Exposure during disposal of infected carcasses.
2000	ND (east)	Farm worker	Infected animal	67, M	Epi-Aid 2000-69	Exposure during disposal of infected carcasses.
1987	Charlotte, NC	Maintenance employee	West Asian cashmere	42, M	Epi-Aid 1987-77	Worked in a goat hair–processing mill.
1978	NH (southeast)	Worker at goat hair– processing mill	Goat hair	20, M	Epi-Aid 1978-65	Loaded hair-carding machine and performed other tasks.
				19, M		Worked at hair mixing and carding machines during week before onset of symptoms.
1978	Shelby, NC	Maintenance worker at goat hair–processing mill	Goat hair	59, M	Epi-Aid 1978-47	
		Temporary worker at goat hair–processing mill	Goat hair	67, M		
1974	Belton, SC	Employee at textile mill	Goat hair	38, F	Epi-Aid 1974-77	Worked in mill spinning area.
1974	Haiti; FL	Navy journalist-photographer	Goatskin in Haitian handicrafts	22, F	Epi-Aid 1974-96	Cutaneous anthrax in FL resident after purchase of *B. anthracis–*contaminated goatskin drums in Haiti.
1971	Gonzales, LA	Two veterinarians	Infected cow	52, M; 26, M	Epi-Aid 1971-131	Disease contracted during necropsy.
1968	Inyo County, CA	Farmhand	Unknown	63, M	Epi-Aid 1969-20	Suspected human cutaneous case, in region of horsefly bite; patient responsible for burning cattle carcasses. Cattle and horsefly exposures considered.
1966	Manchester, NH	Truck driver	Goat hair	35, M	Epi-Aid 1967-43	Truck driver helped unload delivered bales despite being instructed not to help.
1966	Manchester, NH	Unknown	Not determined	35, F	Epi-Aid 1967-43-3	Source uncertain;three samples from hand-knitted sweater positive for *B. anthracis.*
1965, 1969, 1975	Camden, NJ	Three gelatin manufacturing plant workers	Contaminated dry cow bones, used in manufacturing process	29, M; 45, M; ?, M	(41–43)	OSHA fined gelatin factory owners for failure to protect workers.
1964	Oxford, OH	Pipe insulation installer	Goat hair in pipe insulation	36, M	(51)	Fatal cutaneous case featured in a 1965 New Yorker article by Berton Roueche (52).
1960	SC	Four textile mill employees	Goat hair	?	Epi-Aid 1960-31	
1959	Brownsville, Cameron County, TX	Three veterinarians	Necropsy, livestock exposure	?, M; ?, M; ?, M	Epi-Aid 1960-12	One veterinarian had performed necropsy on a steer; other exposures not specified.
		Employee at rendering plant	Not specified	?, M		
		Unspecified	Infected steer	"adolescent boy"		Suspected exposure while skinning steer in Mexico.
1959	NJ (south)	Farmer	Undetermined	23, M	Epi-Aid 1959-38	Possible sources included cows that died of anthrax, and fertilizer with contaminated goat hair.
1957	Vinita, OK	Veterinarian	Infected cow	?, M	Epi-Aid 1958-11	Had performed necropsy on a cow.
1957	Manchester, NH	Two weavers and two card tenders at textile mill	Goat hair	50, F; 64, F; 35, M; 61, M	Epi-Aid 1958-18	
1956	Monroe, NC	Five textile mill employees	Goat hair	?	Epi-Aid 1956-29	
1953	Monroe, NC	Textile mill employee	Goat hair	36, F	Epi-Aid 1953-14	

^a^CDC, Centers for Disease Control and Prevention; OSHA, Occupational Safety and Health Administration. ^b^See Table 2 for additional references. ^c^Excludes investigations in Paraguay and Kazakhstan, where number of human cases uncertain.

Routes of infection were largely a function of setting. Of the 27 cases in textile mills, 21 (78%) were cutaneous, and 6 (22%) were inhalational. Contaminated goat hair or wool was the primary vehicle of infection. Persons working with raw, unprocessed materials were at greatest risk for infection (4). Of the six inhalational cases in textile mills, five were fatal. Three cases of fatal inhalational anthrax were also reported in nontextile mill workers (12,31) (Epi-Aid 1967-43).

Of the 24 investigations in agricultural settings, 9 (38%) included at least one human case. All human cases were acquired cutaneously while a person was handling, performing necropsy on, or disposing of dead animals. The most extensive cross-infection between animals and humans occurred in the 1998 outbreak in Kazakhstan, in which at least 53 human cases occurred; most were cutaneous cases acquired from slaughtering animals (Epi-Aid 1998-83).

Although four investigation reports included concern over possible waterborne transmission ([15] and Epi-Aids 1966-12, 1975-6, 1979-95), this route was not identified in any of the reports of human cases, and water contamination was not regarded as a source of infection. However, disease incidence in animals usually coincided with extremes of wet and dry weather conditions.

Gastrointestinal anthrax was documented in one investigation. Of the 53 persons with anthrax in the 1998 Kazakhstan outbreak, 2 were diagnosed with gastrointestinal anthrax after eating contaminated raw meat. In 1968 in Connecticut, 204 kg of *B. anthracis–*contaminated meat was sold as hamburger before the epizootic investigation; although purchasers of the meat could not be located, no human cases of anthrax were known to have occurred from the contaminated meat (Epi-Aid 1968-78). In addition, in 2000, a Minnesota farm family ate well-cooked meat from a *B. anthracis*–infected steer. Some family members had gastrointestinal symptoms, but investigators could not confirm or rule out infection with *B. anthracis* (54).

### Human Prophylaxis

In nine outbreaks, 136 persons were documented to have received antibiotic postexposure prophylaxis. In at least five of the investigations, postexposure prophylactic therapy was stopped once additional information about risk became available. None of these reports described subsequent infections in patients who received any prophylaxis. Early prophylactic regimens used penicillin injections, which were later replaced by tetracycline, then doxycycline and quinolones, administered orally or parenterally (Epi-Aids 1966-18, 1999-25). Prophylactic antibiotics have been recommended in specific cases involving direct physical contact with contaminated material but are not routinely recommended because the risk for an adverse drug reaction may exceed the risk for infection (Epi-Aid 1975-6). In one report describing a series of events not consistent with public health recommendations, a worker who was potentially exposed to *B. anthracis* in a rendering plant placed a large bottle of tetracycline on a lunchroom table, and coworkers took various amounts of antibiotics if concerned about potential exposure (Epi-Aid 1979-95).

In a 1962 field investigation, an acellular anthrax vaccine was demonstrated to be 93% effective in reducing the risk for infection with *B. anthracis* in humans*.* The vaccine was subsequently recommended for persons who handle imported hair, wool, hides, or bone meal (2).

### Occupational Exposures

In 23 of the 27 U.S. investigations involving human anthrax, exposures occurred in occupational settings. The other four investigations involved exposure to contaminated commercial products or to aerosolized *B. anthracis* spores while a person was passing close to contaminated industrial mills. Among persons exposed in textile mills, most affected workers had direct contact with wool and goat hair as part of their job. However, in 1961, fatal inhalational anthrax occurred in a secretary at a goat hair–processing mill (Epi-Aid 1961-40), and in 1966, cutaneous anthrax occurred in a truck driver who helped unload baled goat hair at the mill (Epi-Aid 1967-43).

In agricultural settings, most cases were in ranchers or other workers who were exposed during the slaughter, butchering, or disposal of *B. anthracis–*infected animals. During 1957–1971, cutaneous anthrax occurred in six veterinarians after they performed necropsies on infected animals; one veterinarian had not used gloves during the necropsy, another had an anthrax lesion on his wrist (suggesting it was uncovered), and no information is available about glove use by the other veterinarians. Other occupational exposures include the goat hair exposures of a pipe insulator in Ohio (51,52) and a weaver in California (12).

### Environmental and Clinical Testing

Specific environmental sampling methods were described in 26 (59%) of the 44 investigations. Sampling methods varied by setting. In textile mills, investigators usually tested samples from raw and processed materials, especially goat hair and wool. In nine investigations, air and surface samples were also tested from numerous locations in and around the mills. In 1978 in North Carolina (Epi-Aid 1978-47), 300 soil samples were taken from the mill premises, the landfill, and private residences near the mill; none tested positive for *B. anthracis*. Samples were also tested from floor sweepings and vacuum cleaner contents from inside the homes of four mill workers; one sample tested positive for *B. anthracis*. In 1953 in North Carolina (Epi-Aid 1953-14), two guinea pigs and four mice were exposed to the air near operating machines in the mill for 3½ hours; no test results are available. No reports of the subsequent investigations of textile mills mentioned the use of such animal tests for environmental sampling during an acute epidemic, although primates were experimentally exposed to air from a *B. anthracis*–contaminated textile mill in South Carolina (55).

In agricultural settings, investigators frequently tested samples of soil, water, and animal carcasses. Environmental sampling was specifically mentioned in 13 agricultural investigations. Elaborate systematic sampling strategies for soil were sometimes used, such as in Louisiana in 1971 (Epi-Aid 1971-131) and in Texas in 1974 (Epi-Aid 1975-6). In other investigations, objects that tested positive for *B. anthracis* in farm settings included hay in Pennsylvania in 1971 (Epi-Aid 1972-19), biting flies in Louisiana in 1955 (Epi-Aid 1955-5), and swine feed made from *B. anthracis*–contaminated bonemeal in Ohio in 1952 (Epi-Aid 1952-13).

During a series of anthrax threats and hoaxes in 1998 (38) (Epi-Aid 1999-25), samples from mailed letters were tested for *B. anthracis* spores by phase microscopy in a university microbiology laboratory, cultured for *B. anthracis* in Laboratory Response Network Level B laboratories (56), and subjected to rapid antigen testing by the U. S. Army Medical Research Institute for Infectious Diseases. All samples from letters were negative. Environmental samples taken from buildings after telephoned threats of contaminated air-handling systems were also negative. In other investigations, objects tested for *B. anthracis* were goat hair pipe insulation (52), imported yarn (12), a knitted sweater (Epi-Aid 1967-43-3), goat hair from contaminated horse saddle pads (44), and Haitian goatskin handicrafts at various stages of the manufacturing process (46,47) (Epi-Aid 1974-96).

With regard to clinical testing in human cases, most detailed reports mention smears and cultures being done on skin lesions and blood samples. Some of these tests were conducted after antibiotics had been started, thereby reducing the likelihood of a positive result. Several of the more recent investigations included serologic tests for antibodies to *B. anthracis* antigens but did not assess the utility of these clinical assays. Nasal swabs were collected from 37 workers during a 1953 North Carolina textile mill anthrax investigation (Epi-Aid 1953-14); laboratory results are not available. No other investigations mentioned use of nasal swabs, and the effectiveness of nasal swabs in detecting *B. anthracis* infection was not discussed in the reports reviewed.

### Decontamination

Several reports recommended specific measures for decontaminating affected areas or materials. A 1953 report suggested that all dirt, dust, and sweepings from a potentially contaminated textile mill be burned (Epi-Aid 1953-14). A 1960 report indicated that a livestock rendering plant was “cleaned up in the recommended manner with 5% hot lye solution” (Epi-Aid 1960-12). A 1967 report recommended installation of a high-temperature furnace at the textile mill for burning wastes (Epi-Aid 1967-43). A 1978 report recommended that potentially contaminated textile mill wastes be soaked in a 5% formaldehyde solution before burial in a landfill (Epi-Aid 1978-65).

The report on Epi-Aid 1972-94 contains the most detail on building-decontamination procedures. In this investigation, an unoccupied New Hampshire textile mill complex slated for demolition was decontaminated. Recommendations were based in part on experience in the earlier decontamination of two South Carolina mill buildings (28); those buildings were subsequently used by another industry for >2 years without any cases of human anthrax being reported. The New Hampshire mill buildings were decontaminated with 9,691 L of liquid formaldehyde that was vaporized and delivered into the interior rooms of the sealed buildings. None of 260 spore strips containing *B. anthracis*, *B. globigii* (now known as *B. atrophaeus),* or *B. subtilis* placed in treated areas of the mill complex showed growth; 23 of 40 such strips placed in untreated (control) areas showed spore growth. In addition, 2 of 555 surface swabs tested positive before treatment, but none of 599 swabs tested positive after treatment. These data from spore strips and surface swabs suggest that the decontamination process was effective in reducing and possibly eliminating the environmental contamination with *B. anthracis*.

During a 1974 anthrax epizootic in Texas (Epi-Aid 1975-6), investigators evaluated the disposal of infected animal carcasses by burning them with old tires, wood, and crank case oil. All 21 samples of carcass ashes, underlying soil, and soil up to 1 m from the burn site were negative for *B. anthracis*.

### Cross-Contamination

Two reports mentioned evidence of cross-contamination from a primary contaminated object to another object or site. In a North Carolina textile mill in 1987 (Epi-Aid 1987-77), investigators speculated that the sample of *B. anthracis*–contaminated Australian wool had been cross-contaminated by *B. anthracis–*contaminated West Asian cashmere stored in the same room. During another North Carolina anthrax outbreak in 1978 (Epi-Aid 1978-47), one of four vacuum cleaner dust samples from the homes of textile mill workers was positive for *B. anthracis*, suggesting that workers carried spores on their clothes from the mills to their homes. No cases of anthrax in workers’ families were reported, suggesting that exposures to *B. anthracis* in the home were not clinically significant.

### Misidentification of Cutaneous Anthrax

A complete differential diagnosis of the clinical manifestations of anthrax includes many other diseases (57,58). In five investigation reports and one MMWR case report, cutaneous lesions initially diagnosed as possible anthrax were subsequently attributed to other diseases (Table 2). In 1975, anthrax was initially suspected in a 23-year-old Arizona man, but his illness was quickly determined to be plague (Epi-Aid 1975-115). In 1973, two sisters in California developed vesiculopapular lesions on their fingers after contact with ill lambs. Anthrax was suspected, but the cultures were negative, and the disease was diagnosed as human orf (59). In 1969, investigators determined that a gram-positive spore-forming bacillus from a skin lesion on a Wyoming meat-packing company worker was not *B. anthracis*, but no definitive species identification could be made (Epi-Aid 1969-78). *B. anthracis* was initially suspected as the cause of cutaneous lesions in persons in a remote village in Nepal in 1967, but plague was subsequently documented (Epi-Aid 1968-34). In 1965, laboratory samples from a skin lesion of a South Carolina customs inspector who had had contact with imported wool were negative for *B. anthracis*. Although no definitive diagnosis was made, the clinical picture made anthrax unlikely (Epi-Aid 1966-18). Finally, in 1957, cutaneous lesions on five New York butchers initially considered as possible anthrax were subsequently diagnosed as pyoderma caused by staphylococci, streptococci, or both (Epi-Aid 1958-16).

## Recommendations and Impact of Investigations

Field investigation reports usually contain public health recommendations; many of these are appropriate for future anthrax epidemics or exposures. For infections associated with textile mills, a 1974 report stated that “decontamination of the primary source of *B. anthracis* is not generally held to be practical” (Epi-Aid 1974-77). The reports on textile mill investigations recommended anthrax vaccine with annually scheduled booster inoculations for mill workers at risk; use of personal protective equipment including specific work clothing and respirators, shower facilities, and separate lockers for work and street clothing; physical separation of raw and finished materials to prevent cross-contamination; design of work areas for easy cleaning; and air-exhaust systems designed to prevent the spread of spores. One report recommended that mill employees be “thoroughly indoctrinated” on the cause, nature, and control of anthrax (Epi-Aid 1953-14). In 1999, following multiple bioterrorist threats (38) (Epi-Aid 1999-25), antibiotic prophylaxis was recommended in cases with known or credible risk for direct exposure. For persons with suspected exposure to aerosolized spores, recommendations included isolating exposed clothing in a plastic bag, showering with copious amounts of soap and water, and washing all possibly contaminated materials with a 1:10 bleach dilution (38).

For infections associated with farms and livestock, reports recommended vaccination of animals at risk, better education of farm workers on anthrax diagnosis and control, thorough destruction by burning of infected animals, prevention of infected livestock from reaching the market, improved supervision of slaughter and meat inspection, and, in some situations, farm quarantine. After the 1974 Texas epizootic (Epi-Aid 1975-06), anthrax vaccine was tested in dairy cattle to assure that the vaccine had no adverse effect on milk safety (6).

Investigations of *B. anthracis–*contaminated saddle pads (1974), Haitian handicrafts (1974), and imported yarn (1976) led to Consumer Product Safety Commission recommendations for destroying those products (7,39,45). In 1975, cutaneous anthrax developed in a New Jersey gelatin manufacturing plant worker after his exposure to contaminated dry cattle bones; the Occupational Safety and Health Administration levied fines for workplace safety violations (41).

The Haitian investigation also led to a federal ban on importing Haitian goatskin products. A review of such handicrafts collected at U.S. quarantine stations in 1980–1981 found that items continued to be contaminated with *B. anthracis* (47). Recommendations to the Haitian Ministry of Health included providing incentives for reporting diseased animals, improving laboratory diagnostic capacity, increasing anthrax vaccination levels among livestock, educating livestock owners about the benefits of anthrax control, and improving the tanning procedures for goatskin drum heads (Epi-Aid 1974-96).

## Discussion

In this report we review what has been learned from >40 epidemiologic field investigations of confirmed or suspected anthrax outbreaks in humans or animals during the last 50 years. In the 2001 bioterrorism response, investigators evaluated suspected anthrax cases by using clinical and laboratory diagnostic methods, such as chest radiographs, cultures, and serologic assays, that had been developed and refined during earlier investigations of inhalational and cutaneous anthrax in textile mill workers. In addition, histopathologic and immunohistochemical testing proved essential for diagnosing anthrax in persons who had been placed on antibiotics early and whose cultures were thus negative. Nasal swabs, as used in the 1953 textile mill investigation, are currently considered an unevaluated adjunct to environmental sampling for defining exposed populations in bioterrorism investigations (1,60). Nasal swabs were used in the 2001 investigation for defining the aerosol spread of *B. anthracis* spores in the Hart Senate Office Building and some other settings.

In the recent investigation, an anti-protective antigen, enzyme-linked immunosorbent assay (61) was used to confirm *B. anthracis* infection in several cases. Development of this assay was the culmination of decades of laboratory experience and research associated with past field investigations of anthrax.

Asymptomatic infection was documented in one serologic survey (33) conducted several months after an inhalational anthrax outbreak; however, in past and current investigations, the role of asymptomatic infection in providing protection is unclear. Human-to-human spread was not evident in any of the investigations reviewed.

Investigation into a series of anthrax-related threats and hoaxes in 1998 (Epi-Aid 1999-25) also helped lay the groundwork for the recent response. In that investigation, guidelines for risk assessment and postexposure antibiotic prophylaxis were developed, and coordination with first responders and law enforcement was emphasized (38). The investigation also led to revised immunization recommendations (5), which discuss the use of vaccine for postexposure prophylaxis*.*

In response to the bioterrorism events of 2001, additional guidelines were published on investigating and responding to *B. anthracis* exposures. These address clinical testing, use of antibiotic prophylaxis, closing of potentially contaminated buildings, and postexposure treatment options (1,62,63). Current recommendations for the use of anthrax vaccine are based in large part on a field trial conducted in 1962 (2,5). During the 2001 response, vaccination recommendations were expanded to at-risk populations; the 1962 vaccine efficacy study forms part of the justification for considering the vaccine for postexposure prophylaxis. Currently, the Advisory Committee on Immunization Practices recommends that vaccine be used in combination with antibiotics (ciprofloxacin, doxycycline, or penicillin) following a *B. anthracis* bioterrorism exposure, if vaccine is available (5). Vaccination is a critical component of the nation’s preparedness and response activities for *B. anthracis* bioterrorism.

In past field investigations, the primary risk factor for human cutaneous anthrax has been direct physical contact with infected animals or commercial products containing *B. anthracis* spores. Ranchers, butchers, and veterinarians were at risk for such contact when working with infected animals. All the commercial products causing human infection were of animal origin; most were made from imported goat skin or hair.

For inhalational anthrax, the main risk factor was exposure to aerosolized spores, especially in or near a textile mill that processes goat hair. While it is unclear why some workers become infected while others in the same dusty environment do not, several factors may increase the likelihood for infection. First, direct work with unprocessed goat hair may create a heavier exposure to *B. anthracis* spores. Second, a weakened immune system may increase a person’s susceptibility to infection (64). Two of the patients with inhalational anthrax probably had chronic pulmonary disease. In the 1957 investigation, sarcoidosis was present (31). In the 1966 investigation of a metal shop worker (Epi-Aid 1967-43), investigators noted the worker’s “chronic cigarette cough” and suggested that his alcoholism, diabetes, and pancreatitis might have made him more susceptible than his healthy coworkers.

Over the past 50 years, a series of recommendations have focused mainly on preventing occupationally acquired anthrax, especially in textile mills and agricultural settings. For example, in 1962, anthrax vaccine was recommended for persons who handle imported hair, wool, hides, or bonemeal (2). More recently, it was recommended that veterinarians obtain diagnostic specimens but not perform necropsies on animals suspected to have died from anthrax (36). The National Institute for Occupational Safety and Health has been actively involved in many recent anthrax-related investigations (15,65).

Some documents mentioned insects as possible vectors in the spread of *B. anthracis*. While mechanical spread of *B. anthracis* organisms by stable flies has been demonstrated in guinea pigs (66), the importance of insects as vectors in epizootics has not been determined. One hypothesis suggests that insect bites might allow superficial organisms an effective access point for intradermal infection. Insects, particularly horseflies, were explicitly mentioned in 12 investigations for their possible role in transmission; however, no evidence exists that biting flies contribute to transmission of disease from animals to humans.

Past methods for decontaminating buildings relied upon formaldehyde gas, now known to be carcinogenic. The recent decontamination of *B. anthracis–*contaminated buildings was accomplished with chlorine dioxide gas, by using the methods developed for decontaminating textile mill buildings. Pre- and posttreatment environmental sampling strategies developed in several of the earlier field investigations, including the systematic use of surface swabs and spore strips, were also used in the response to recent events. In these events, the wide dispersion from envelopes of small airborne particles containing spores led to higher than expected levels of cross-contamination, making decontamination more difficult (65).

Several limitations should be considered in interpreting the results of this review. CDC conducts field investigations only when invited by a state health department or ministry of health. Anthrax cases that did not actively involve CDC staff, such as those investigated solely by state or local health departments, were excluded; therefore, this is not a complete report of U.S. anthrax case investigations. However, CDC staff have consulted at least by telephone on almost every case of human anthrax reported in the United States since the 1950s (A. Kaufmann, pers. comm.). A manuscript reviewing the characteristics of all anthrax cases reported in the United States since 1955 is in preparation (D. Ashford, pers. comm.). Second, this review examines CDC’s experience with field investigations involving anthrax; laboratory-based anthrax research was not included unless it was related to a field investigation. Third, final laboratory results were not available for some field investigations.

## Conclusion

Much useful knowledge, ranging from the diagnosis of anthrax to the use of vaccine to protect populations, has been gained from these past investigations. However, many questions remain. Further research is needed to determine the lowest infectious dose, define what constitutes a true exposure for which antibiotic prophylaxis is warranted (especially in light of possible drug side effects), and determine whether spores delivered in an envelope create a residual risk after the primary contamination event. Other areas in which more research is needed include developing better rapid environmental testing methods (67), identifying optimal decontamination methods for a variety of contaminated settings, assessing *B. anthracis* spore background rates in selected settings, and determining the level of risk associated with a low degree of exposure to aerosols containing *B. anthracis*.

During the past 50 years, the scientific knowledge acquired in these field investigations has greatly improved the nation’s ability to respond to anthrax outbreaks. New and unique challenges have been raised by the recent intentional release of *B. anthracis*. Further efforts to improve knowledge about anthrax, both in its natural setting and in the context of bioterrorism, are urgently needed.
